# A Swift Solution: Early Removal of the JADA Device in the Management of Postpartum Hemorrhage

**DOI:** 10.7759/cureus.104570

**Published:** 2026-03-02

**Authors:** Heather Wang, Leshae Cenac, Alicia Huckaby

**Affiliations:** 1 Department of Obstetrics and Gynecology, Mercy Health St. Vincent Medical Center, Toledo, USA

**Keywords:** early removal, jada device, obstetric emergency, postpartum hemorrhage, uterine atony

## Abstract

Postpartum hemorrhage (PPH) continues to be a major threat to maternal health worldwide. Current management strategies include uterotonic agents, intrauterine tamponade devices, and surgical interventions. These methods have variable efficacy, carry potential complications, and contribute to significant healthcare costs. The JADA device is an emerging and promising tool for managing PPH. We report the case of a 19-year-old G2P1011 at 39w3d who experienced uterine atony following a spontaneous vaginal delivery. Despite administration of Pitocin and tranexamic acid, the patient continued to have brisk vaginal bleeding after placental delivery. She was subsequently transferred to the OR for an exam under anesthesia due to her intolerance of pelvic examinations. Uterine atony was confirmed, and a second-degree perineal laceration with minimal bleeding was noted. The decision was made to place a JADA device when the bleeding did not resolve with uterotonics and bimanual massage. The second-degree perineal laceration was repaired in standard fashion. The JADA device was successfully removed after 30 minutes, rather than the manufacturer-recommended one-hour duration. Total quantified blood loss was 1300 mL. The patient was stable for discharge on postoperative day 2 and had an uncomplicated postoperative and postpartum course despite early removal of the JADA device. This case highlights the efficacy and safety of early JADA removal in the management of PPH. Recent data suggest that the JADA offers distinct advantages over traditional tamponade devices such as the Bakri balloon. The device’s versatility may be particularly beneficial in settings where prolonged placement is not feasible. Beyond obstetrics, the JADA device has shown potential in gynecologic procedures, such as after myomectomies. Overall, the JADA device offers a safe, effective, and versatile tool for PPH management. This case contributes to the growing evidence supporting its role in individualized care and underscores the need for further studies exploring early removal protocols.

## Introduction

Postpartum hemorrhage (PPH) remains a significant cause of maternal morbidity and mortality worldwide. According to the World Health Organization, approximately 27% of maternal deaths in low- and middle-resource countries and 16% of maternal deaths in high-resource countries are attributed to PPH [1]. The U.S. has one of the highest rates of maternal mortality, with PPH contributing to about 8% of all causes [2]. The American College of Obstetricians and Gynecologists defines PPH as cumulative blood loss greater than or equal to 1000 mL within 24 hours of delivery, regardless of the route of delivery. Despite advances in obstetric care, managing PPH continues to pose challenges, emphasizing the need for effective, innovative, and cost-efficient interventions to improve maternal outcomes.

Traditional approaches to PPH management consist of uterotonic agents, intrauterine tamponade devices, and surgical interventions. Common uterotonic agents include oxytocin (Pitocin), methylergonovine (Methergine), carboprost (Hemabate), and misoprostol (Cytotec). When medical management fails, mechanical tamponade devices are used, including the Bakri balloon. These devices function by exerting outward pressure against the uterine walls to compress bleeding blood vessels. They require inflation with large fluid volumes, may contribute to uterine distention, and can be associated with patient discomfort or persistent bleeding if tamponade is inadequate. Additionally, these methods have variable efficacy, carry potential complications, and contribute to significant healthcare costs [3].

The JADA device, a vacuum-induced uterine tamponade device, is an emerging and promising tool in PPH management. It was approved by the U.S. Food and Drug Administration in 2020 for the control and treatment of PPH. Unlike traditional tamponade devices that rely on outward mechanical pressure, the JADA uses negative intrauterine pressure via continuous wall suction to stimulate physiologic uterine contractions and evacuate retained blood from the uterus. The device consists of a soft silicone intrauterine loop with vacuum pores and a cervical seal balloon that stabilizes placement when inflated with saline. Studies have demonstrated JADA’s advantages over traditional methods in clinical outcomes [4].

Recent literature has highlighted the potential advantages of the JADA device compared with conventional uterine balloon tamponades such as the Bakri balloon. Research comparing the two devices suggests that the JADA may be more cost-effective. Bridges et al. demonstrated that using the JADA device for PPH not only leads to fewer complications, specifically, the need for additional procedures, ICU admissions, and maternal deaths, but also reduces healthcare costs compared with Bakri balloons [5]. The JADA device has also been associated with reductions in massive transfusions and overall estimated blood loss compared with uterine balloon tamponades like the Bakri balloon [6]. Additionally, adoption of the JADA system has been shown to reduce healthcare expenses while maintaining high safety standards and reducing resource consumption [7]. While some of these findings are derived from observational and modeled analyses rather than randomized controlled trials, the available data suggest potential clinical and economic advantages in selected patient populations.

This case report highlights the use of the JADA device in the management of PPH, emphasizing the safety and effectiveness of early device removal without increasing the risk of adverse maternal outcomes. Although manufacturer guidelines recommend maintaining suction for approximately one hour, this report explores individualized removal following confirmation of sustained hemostasis. Early removal may be advantageous in select patients when prolonged device retention presents clinical, anesthetic, or logistical challenges. By describing objective criteria supporting early removal, this report contributes to the growing evidence surrounding optimal JADA device retention time and individualized PPH management.

## Case presentation

Our patient was a 19-year-old G2P1011 female who delivered at 39w3d after presenting to Labor and Delivery for a risk-reducing induction of labor. She subsequently developed acute PPH, characterized by brisk vaginal bleeding and persistent uterine atony, requiring placement of a JADA device.

On arrival, the patient reported regular contractions and leakage of fluid for three days. She was unable to tolerate a cervical examination due to a history of trauma and pain from contractions. However, she was noted to be grossly ruptured with clear fluid. She received augmentation with Pitocin and artificial rupture of membranes of a forebag. She ultimately received an epidural for pain control, but she remained intolerant of pelvic examinations due to painful contractions despite repeat epidural boluses. The patient also experienced significant anxiety, which required extensive emotional support from the medical team.

She progressed to complete cervical dilation and delivered a viable infant via spontaneous vaginal delivery. After placental delivery, brisk vaginal bleeding was noted. A Pitocin bolus was administered, and tranexamic acid (TXA) was given in response to ongoing bleeding. Vital signs remained stable at this time. Initial quantified blood loss (QBL) was approximately 500 mL. Fundal examination revealed a boggy and enlarged uterus, consistent with uterine atony.

Given continued brisk bleeding despite administration of uterotonics and the patient’s inability to tolerate an adequate bedside examination, a thorough assessment for additional sources of hemorrhage could not be safely performed. The decision was made to proceed to the OR for further evaluation and management. The patient verbally consented to an exam under anesthesia (EUA), possible repair of vaginal or cervical lacerations, and control of PPH. To expedite care, an overhead obstetric hemorrhage rapid response was activated. She was then transferred to the OR and received 2 g of Ancef intraoperatively for antibiotic prophylaxis.

**Figure 1:**
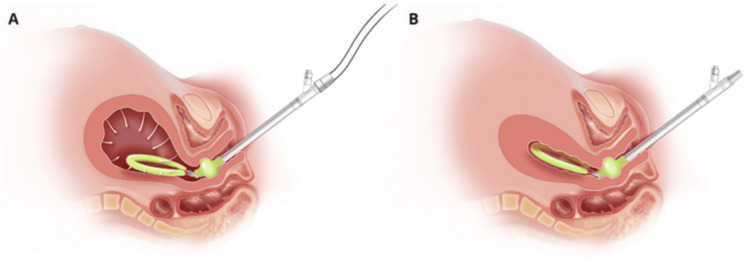
(A) JADA with continuous wall suction being applied; (B) JADA with uterine compression due to negative intrauterine pressure Image credit: Organon (2022) [8]

Under general anesthesia, a sterile vaginal preparation was performed. EUA revealed a second-degree perineal laceration with minimal bleeding, absent cervical lacerations, and persistent uterine atony despite bimanual massage and prior administration of Pitocin and TXA. The decision was made to place a JADA device for further control of uterine atony. The device was inserted per manufacturer guidelines, with the cervical seal balloon inflated with 120 mL of saline and connected to continuous wall suction of 80 mmHg (Figure 1).

Because the patient had previously been unable to tolerate pelvic examinations and there was concern about safely removing and assessing the JADA device postoperatively, the decision was made to maintain active intraoperative surveillance for 25 minutes under general anesthesia. No additional blood was observed in the suction canister during this time. Her uterine fundus was firm and located 3 cm below the umbilicus. Given stable bleeding, continuous suction was discontinued, and 120 mL of saline was evacuated from the cervical seal. An additional five minutes of monitoring showed no further bleeding. The patient’s uterine fundus remained 3 cm below the umbilicus with appropriate tone. A final bimanual examination confirmed resolution of uterine atony. The second-degree laceration was repaired in standard fashion using 3-0 Vicryl. The patient was then extubated and transferred to the recovery unit in stable condition. Total QBL after the procedure was 1300 mL.

Her postpartum and postoperative course was uncomplicated, with no further episodes of abnormal vaginal bleeding. She remained hemodynamically stable and was discharged from the hospital on postpartum day 2. Her six-week postpartum follow-up remained uneventful despite early removal of the JADA device.

## Discussion

PPH remains a global challenge in obstetric care. There has been a rise in PPH cases, accompanied by an increase in interventions such as hysterectomies and uterine artery embolizations across the U.S. from 2001 to 2012 [9]. While traditional management strategies, including uterotonic agents, intrauterine tamponade devices, and surgical interventions, have played a critical role in addressing PPH, these approaches have limitations. Notable concerns include delayed uterine contraction, uterine overdistension from balloon inflation, risk of infection, patient discomfort, and failure requiring escalation to invasive procedures. Research comparing intrauterine tamponade devices and uterine artery embolization found no significant difference in the risk of peripartum hysterectomy and/or maternal death between the two methods [10], suggesting that less invasive options may be equally effective in certain scenarios. Furthermore, higher QBL during delivery has been shown to correlate with longer hospital stays and increased healthcare costs. Khan et al. reported that for each additional 100 mL of blood loss during a vaginal delivery, there was a 2.4% increase in hospital length of stay and a 1.3% increase in healthcare costs, highlighting the financial implications of PPH [11]. To address these limitations, more effective and innovative solutions are needed.

The JADA device has emerged in recent years as a promising alternative to conventional methods for managing PPH. By mimicking the physiology of the postpartum uterus and using negative intrauterine pressure to promote uterine contractions and control bleeding, the JADA device addresses some of the shortcomings of traditional uterine tamponade devices. Unlike balloon systems that rely on outward mechanical compression to tamponade bleeding vessels, the JADA device promotes active uterine contraction through a vacuum, which enhances passive uterine compression. This mechanism may allow for rapid control of persistent bleeding due to uterine atony.

When compared to the Bakri balloon, the JADA device offers several additional advantages, including reductions in the rates of blood transfusions, ICU admissions, uterine artery embolizations, and hysterectomies [12]. Specifically, the proportion of patients requiring ≥4 units of packed red blood cells was significantly lower in patients using a JADA device compared with those using an intrauterine balloon tamponade. In a theoretical cohort of 75,000 women, use of the JADA device resulted in 4,836 fewer blood transfusions, 7,388 fewer additional surgical procedures, 5,176 fewer ICU admissions, 143 fewer maternal deaths, and an annual cost saving of $145 million [5]. Cost-effectiveness analyses further highlight the economic advantage of the JADA device [13]. These attributes may make the device particularly valuable in resource-limited settings, where safe, efficient, and cost-effective management of PPH is crucial.

Beyond its application in PPH, the JADA device has also demonstrated versatility in addressing abnormal uterine bleeding during gynecologic procedures, such as after myomectomies. Lee et al. used the JADA device as an alternative to balloon tamponade to control severe uterine bleeding following a vaginal myomectomy of a large prolapsing necrotic myoma [14]. The device was successfully removed on postoperative day 1 without complications. This successful application broadens the utility of the JADA device beyond traditional PPH control.

This case report highlights the successful early removal of the JADA device in the context of PPH, offering a new perspective on its utility. In this patient, the decision to remove the device after 30 minutes rather than the manufacturer-recommended one hour was based on objective intraoperative findings. After 25 minutes of continuous suction, no additional blood was observed in the suction canister. The uterine fundus remained firm and positioned 3 cm below the umbilicus, and repeat bimanual examination confirmed sustained resolution of uterine atony. The patient remained hemodynamically stable, and no cervical or deep vaginal lacerations were identified to suggest an alternative bleeding source. Importantly, the patient’s significant intolerance to pelvic examinations and need for general anesthesia also influenced management decisions. Maintaining active intraoperative surveillance allowed direct confirmation of hemostasis prior to removal, avoiding the need for subsequent bedside manipulation or re-anesthetization.

Early device removal carries a theoretical risk of recurrent bleeding or insufficient uterine contraction, particularly in the absence of data regarding the optimal minimum duration of JADA placement. However, the clearly defined indicators of hemostasis observed in this case supported safe removal. This suggests that individualized device removal may be appropriate in select patients. While a single case cannot establish new standards of care, it contributes to the growing body of evidence supporting the versatility of the JADA device and individualized management strategies for PPH.

## Conclusions

The JADA device has demonstrated promising effectiveness, safety, and potential cost advantages over traditional PPH management strategies. Recent data suggest that its use is associated with reductions in blood transfusions, ICU admissions, and healthcare costs when compared with intrauterine balloon tamponades. Its utility in managing abnormal uterine bleeding further highlights its growing significance in both obstetric and gynecologic settings, such as post-myomectomy hemorrhage. This case report contributes to the evolving understanding of the JADA device’s role in managing PPH by describing successful early removal without immediate compromise to patient safety or clinical outcomes. The device provided a minimally invasive method to resolve uterine atony and control bleeding, thereby avoiding escalation to more invasive surgical interventions.

Although findings from this case do not establish definitive recommendations, this literature review and case report illustrate the potential to adapt the JADA device for individualized use. Further research is warranted to evaluate long-term outcomes, including the risk of recurrent PPH, the need for repeat interventions, and overall maternal morbidity associated with early removal, in order to inform clinical recommendations and further advance the field of obstetrics.
